# Inhibitory Potential of the *Ocimum sanctum* Phytochemicals on Bruton’s Tyrosine Kinase, a Well-Known Drug Target for Treatment of Chronic Lymphocytic Leukemia: An In Silico Investigation

**DOI:** 10.3390/molecules28083287

**Published:** 2023-04-07

**Authors:** Shabir Ahmad Mir, Yahya Madkhali, Ahmad Firoz, Ayoub Al Othaim, Wael Alturaiki, Sami G. Almalki, Abdulrahman Algarni, Suliman A. Alsagaby

**Affiliations:** 1 Department of Medical Laboratory Sciences, College of Applied Medical Science, Majmaah University, Al Majmaah 11952, Saudi Arabia; 2 Health and Basic Sciences Research Center, Majmaah University, Al Majmaah 11952, Saudi Arabia; 3 Department of Biological Sciences, Faculty of Science, King Abdulaziz University, Jeddah 21589, Saudi Arabia; 4 Department of Medical Laboratory Technology, Faculty of Applied Medical Sciences, Northern Border University, Arar 91431, Saudi Arabia

**Keywords:** *Ocimum sanctum*, chronic lymphocytic leukemia, molecular docking, in silico, phytochemicals, cancer

## Abstract

Chronic lymphocytic leukemia (CLL) is an incurable neoplasm of B-lymphocytes, which accounts for about one-third of all leukemias. *Ocimum sanctum*, an herbaceous perennial, is considered as one of the important sources of drugs for the treatment of various diseases, including cancers and autoimmune diseases. The present study was designed to screen various phytochemicals of *O. sanctum* for discovering their potential to inhibit Bruton’s tyrosine kinase (BTK), a well-known drug target of CLL. Various phytochemicals of *O. sanctum* were screened for their potential to inhibit BTK using several in silico protocols. First, the molecular docking approach was used to calculate the docking scores of the selected phytochemicals. Then, the selected top-ranked phytochemicals were screened for their physicochemical characteristics using ADME analysis. Finally, the stability of the selected compounds in their corresponding docking complexes with BTK was analysed using molecular dynamics simulations. Primarily, our observations revealed that, out of the 46 phytochemicals of *O. sanctum*, six compounds possessed significantly better docking scores (ranging from −9.2 kcal/mol to −10 kcal/mol). Their docking scores were comparable to those of the control inhibitors, acalabrutinib (−10.3 kcal/mol), and ibrutinib (−11.3 kcal/mol). However, after ADME analysis of these top-ranked six compounds, only three compounds (Molludistin, Rosmarinic acid, and Vitexin) possessed drug likeliness characteristics. During the MD analysis, the three compounds Molludistin, Rosmarinic acid, and Vitexin were found to remain stable in the binding pocket in their corresponding docking complexes with BTK. Therefore, among the 46 phytochemicals of *O. sanctum* tested in this study, the three compounds, Molludistin, Rosmarinic acid, and Vitexin are the best inhibitors of BTK. However, these findings need to be confirmed by biological experiments in the laboratory.

## 1. Introduction

Chronic lymphocytic leukemia (CLL) is a malignancy of B lymphocytes, which is characterized by continuous growth and proliferation of malignant B lymphocytes in bone marrow, peripheral blood, lymph nodes, and spleen. CLL is the most widespread adult leukemia in Western countries, and it accounts for about 25–30% of all the leukemia. In 2019, over 100,000 incidence cases of CLL were reported worldwide [1,2,3]. However, the incidence of CLL significantly varies based on ethnicity, gender, age, and other genetic and non-genetic factors [1,2]. The CLL incidence is reported to be higher in elderly populations [4] and in males [5,6]. The disease is caused by the overgrowth of a single CD5^+^ B lymphocyte, which co-express low levels of several other surface-markers, including CD23, CD79b, and CD20. The clinical outcomes of this disease vary among different patients. Some CLL patients die within three years, whereas others survive for decades [2]. Additionally, the treatment outcomes of CLL are reported to be very diverse (https://www.lls.org/leukemia/chronic-lymphocytic-leukemia/treatment/treatment-outcomes (accessed on 28 February 2023)). One of the versatile drug targets for discovery of drugs against CLL is the intracellular protein kinase known as Brutons’s tyrosine kinase (BTK). Several BTK inhibitors (BTKIs), including Ibrutinib and Acalabrutinib, are already a part of the currently available chemotherapy for CLL [7,8]. However, improved inhibitors of BTK are of great need due to the limitations associated with the clinically available ones. For example, side effects, such as hematotoxicity, anemia, cytopenias, increased risk of infections, bleeding, hypertension, and atrial fibrillation, have been reported in CLL patients treated with Ibrutinib [9]. Another major issue limiting the beneficial use of Ibrutinib is the resistance developed by CLL cells to the drug [9]. Despite the improvement made in the selectivity of Acalabrutinib compared with Ibrutinib, the adverse events of treatment with Acalabrutinib are similar to those reported in CLL patients treated with Ibrutinib [10,11]. Overall, these limitations of the available BTK inhibitors call for more searches for novel inhibitors that would improve the responsiveness and safety of targeting BTK in CLL patients [7,8].

The rise of cancer incidence, and the limitations of chemotherapeutic treatment, have diverted the attention of researchers towards herbal remedies and newer dimensions are being searched for therapy of cancers, including CLL [12]. Various parts of the medicinal plants have been widely used in traditional medicine to treat various diseases since times immemorial. The efficient screening of plant species with the objective of finding new bioactive compounds is a regular activity in various research laboratories. The identification of the active principles in the plants after a long process of screening and evaluation leads to the development of new/novel drugs against diseases [13]. In fact, various currently available drugs for different diseases have been discovered from the naturally occurring plant sources [13,14]. Further exploration of the chemical constituents of the plants and their pharmacological screening is therefore essential to develop new and improved life-saving drugs for various life-threatening diseases, including different types of cancers [13]. One of the plant herbs, *Ocimum sanctum* L. (also known as Tulsi), has been used in traditional medicine for treating various diseases for thousands of years. Its extracts are used in Ayurvedic remedies for headaches, stomach disorders, common colds, heart disease, inflammation, and various forms of poisoning and malaria [13]. Recently, some of the phytochemicals of *O. sanctum* have been reported to be good inhibitors of various drug targets of SARS-CoV-2 and have been proposed to be possible future drugs for treating SARS-CoV-2 infections [15,16,17]. Various useful effects of *O. sanctum* including anti-inflammatory, analgesic, antipyretic, antidiabetic, hepatoprotective, hypolipidemic, immune modulatory, and anti-stress activity have been reported [13,18,19]. In addition, the anti-cancer properties of *O. sanctum* leaves are well documented [19,20]. For example, the phytochemicals (eugenol, orientin, and vicenin) from leaf extract of *O. sanctum* have been reported to possess anti-cancer activity against breast cancer, liver cancer, and fibrosarcoma [20,21]. Furthermore, the anticancer potential of *O. sanctum*, especially its leaf extracts and seed oil, has been explained by several cell line-based and animal-based studies [13,22,23,24]. However, the role of individual phytochemicals of *O. sanctum* in the treatment of CLL has not been reported. Therefore, the current in silico study has been designed with a rationale to screen the major phytochemicals of *O. sanctum* for their potential to bind and inhibit BTK for the treatment of CLL. This study is one of the preliminary studies to discover new plant derived inhibitors of BTK for the efficient treatment of CLL.

## 2. Results

### 2.1. Molecular Docking

In the present study, 46 phytochemicals of *O. sanctum (Tulsi)* were screened through in silico procedures for their ability to bind and inhibit BTK [PDB ID: 5P9J], a well-recognized drug target for chronic lymphocytic leukaemia (CLL) (Table 1). Our results revealed that, out of the 46 phytochemicals of *O. sanctum*, six compounds (Vicenin-2, Luteolin-7-O-glucuronide, Molludistin, Galuteolin, Vitexin, and Rosmarinic acid) showed a potential binding affinity towards the active site of BTK [PDB ID: 5P9J], as represented by their relatively higher docking scores of more than −9 kcal/mol (Table 1). The docking scores of these six top-ranked phytochemicals (ranging from −9.2 kcal/mol to −10 kcal/mol) were comparable to those of the well-established inhibitors of BTK, Ibrutinib (−11.3 kcal/mol), and Acalabrutinib (−10.3 kcal/mol), which were used as positive control inhibitors in this study (Table 1). The ligand (Ibrutinib) in the original PDB protein structure 5P9J was extracted from the structure and redocked with the empty protein to validate the docking procedure and interestingly we observed a docking score of −11.2 kcal/mol, which was similar to that observed for separately docked Ibrutinib (−11.3 kcal/mol), downloaded from Pubchem (CID 24821094).

### 2.2. Protein–Ligand Interaction Analysis

**Figure 1:**
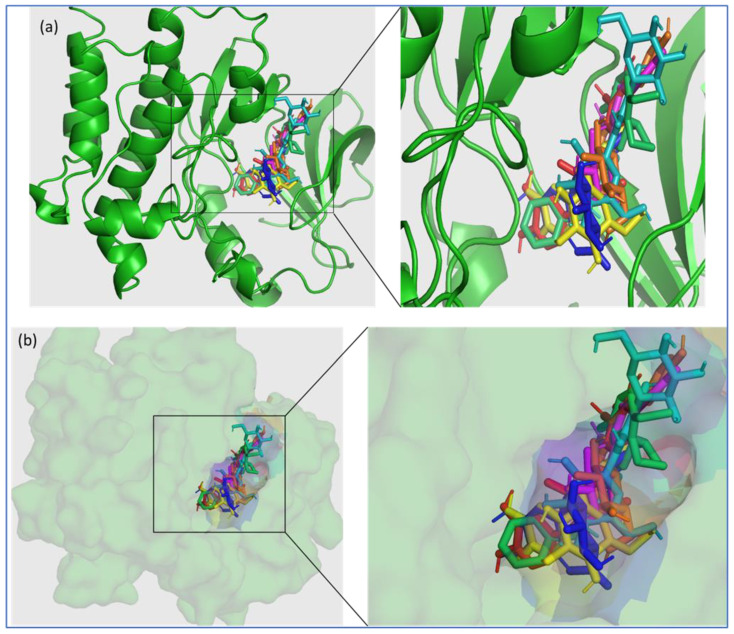
Three-dimensional (3D) illustration of the binding of the top-ranked phytochemicals of *O. sanctum* (Vicenin-2, Luteolin-7-O-glucuronide, Molludistin, Galuteolin, Vitexin, and rosmarinic acid) and the control drug (ibrutinib) to the active site of Bruton’s tyrosine kinase (PDB ID: 5P9J). Bruton’s tyrosine kinase is represented in green color, where the phytochemicals and ibrutinib are shown in separate colors; Ibrutinib (lime green), Vicenin-2 (cyan), Luteolin-7-O-glucuronide (blue), Molludistin (magenta), Galuteolin (yellow), Vitexin (orange), and rosmarinic acid (red). (**a**) Cartoon representation, (**b**) surface view. The insets in (**a**,**b**) show the magnified views of the selected portion of the of BTK active site occupied by the phytochemicals/ligands.

**Figure 2:**
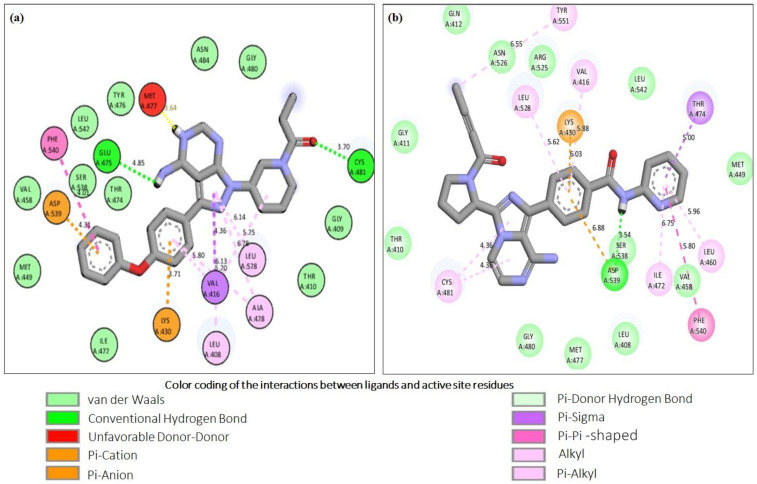
Molecular interaction analysis of the control inhibitors, ibrutinib and acalabrutinib upon docking with BTK. The figure shows the respective two-dimensional illustrations of the interaction of Ibrutinib (**a**) and Acalabrutinib (**b**) with specific amino acid residues in the active site of BTK. The interactions of the control inhibitors with the active site residues of BTK are represented as dotted lines of various colors and their corresponding bond distances are also shown in the figure. The color coding of the interactions is provided at the bottom of the figure.

**Figure 3:**
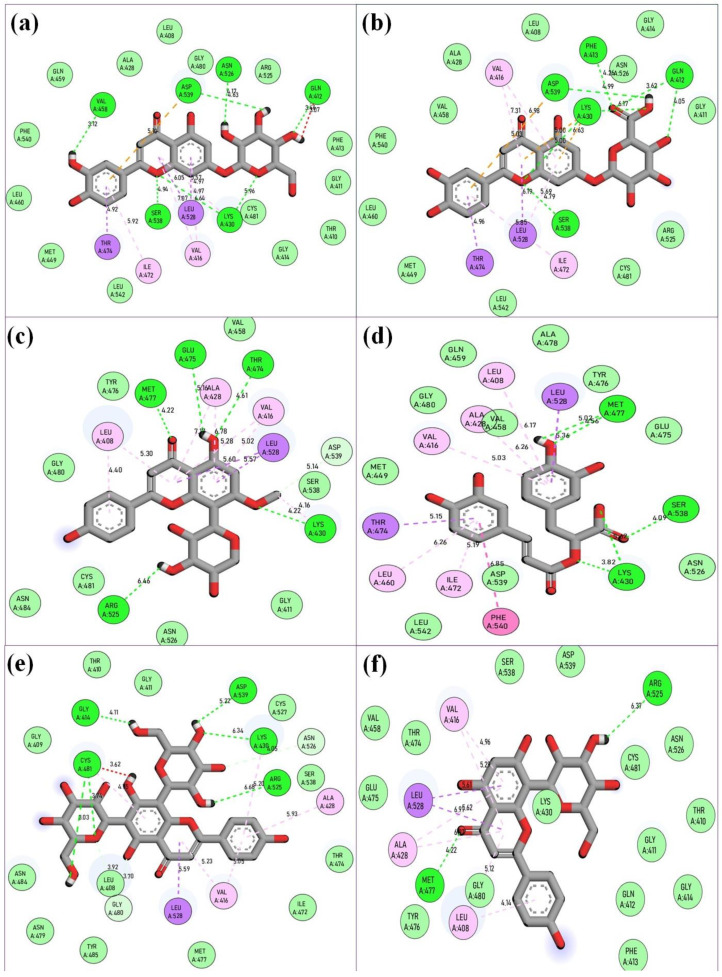
Molecular interaction analysis of the top-ranked phytochemicals of *O. sanctum* (Vicenin-2, Luteolin-7-O-glucuronide, Molludistin, Galuteolin, Vitexin, and rosmarinic acid) upon docking with BTK. The figure shows the respective two-dimensional illustrations of the interaction of the phytochemicals with specific amino acid residues in the active site of BTK. (**a**) Galuteolin, (**b**) Luteolin-7-O-glucuronide, (**c**) Molludistin, (**d**) rosmarinic acid, (**e**) Vicenin-2, and (**f**) Vitexin. The interactions of the selected phytochemicals with the active site residues of BTK are represented as dotted lines of various colors and their corresponding bond distances are also shown in the figure. The color coding of the interactions is same as provided in Figure 2.

Since the higher docking scores of the ligands, as represented by their lower free energy (ΔG) values, proportionally indicate the affinity of the ligand to the receptor protein, therefore, the top-ranked six phytochemicals from *O. sanctum*, viz; Vicenin-2 (−10 kcal/mol), Luteolin-7-O-glucuronide (−9.8 kcal/mol), Molludistin (−9.7 kcal/mol), Galuteolin (−9.6 kcal/mol), Vitexin (−9.3 kcal/mol), and Rosmarinic acid (−9.2 kcal/mol) were selected for studying their interaction with the active site residues of BTK. The best poses of the selected top-ranked phytochemicals of *O. sanctum* upon docking with 5P9J and their location in the binding pocket of BTK are shown in Figure 1 (also see Supplementary Materials Figure S1). It was observed that these top-ranked phytochemicals binded to the active site/binding pocket of BTK at the same location where the control drug and Ibrutinib bind (Figure 1 and Supplementary Figure S1). On performing the interactions studies, it was observed that the top-ranked phytochemicals of *O. sanctum* interacted with the active site amino acid residues of BTK through multiple interactions (hydrogen bonds, hydrophobic interactions, electrostatic interactions, etc.). The two-dimensional (2D) interaction of the best poses of the control drugs and the top-ranked phytochemicals of *O. sanctum*, with the active site residues of BTK, along with the bond lengths of the various types of interactions, are shown in Figure 2 and Figure 3, respectively.

### 2.3. ADME Analysis

In the present study, the ADME analysis of the six top-ranked phytochemicals was performed for further screening and selection of the best compounds possessing drug-like characteristics. The detailed results of ADME/pharmacokinetics prediction analysis of the six top-ranked phytochemicals of *O. sanctum* are shown in Supplementary Table S1. The Swiss-ADME analysis of the top-ranked phytochemicals of *O. sanctum* using the ESOL method [25] predicted all the six phytochemicals (Vicenin-2, Luteolin-7-O-glucuronide, Molludistin, Galuteolin, Vitexin, and rosmarinic acid) as soluble in water, whereas, the Ali method [26,27] predicted the three phytochemicals (Galuteolin, Luteolin-7-O-glucuronide, and rosmarinic acid,) as moderately soluble, and three phytochemicals (Molludistin, Vitexin, and Vicenin-2) as purely soluble in water (Table 2 and Supplementary Table S1). Moreover, on applying the Lipinski rule of 5, we found that, among the six phytochemicals, the physiochemical properties of two compounds (Molludistin and Rosmarinic acid) did not violate any criterion among the set criteria, whereas Vitexin violated only one criterion. The other three phytochemicals, including Galuteolin, Luteolin-7-O-glucuronide, and Vicenin-2 violated two or more criteria (Table 2 and Supplementary Table S1). Based on Lipinski rule of 5 and other ADME analysis filters, out of the six top-ranked phytochemicals, three compounds, viz., Molludistin, rosmarinic acid, and Vitexin were selected for further analysis through in silico toxicity assay and molecular dynamics (MD) simulations.

### 2.4. In Silico Toxicity Analysis

The results of oral acute toxicity and the corresponding toxicity class for each compound are shown in Table 3. The results show that Molludistin and Vitexin are in toxicity class IV with predicted lethal doses of 832 mg/kg body weight (832 mg/kgbw) each, whereas rosmarinic acid lies in class V, with a predicted lethal dose of 5000 mg/kgbw. These results demonstrate the less-toxic nature and safety of these compounds. The organ toxicity results and the calculated predictions for various toxicological endpoints using the ProTox-II web server are reported in Supplementary Table S2. These results showed that all the three tested phytochemicals were predicted as inactive compounds for hepatotoxicity. However, Molludistin and rosmarinic acid were predicted as active compound for immunotoxicity endpoint, whereas Vitexin was predicted as an active compound for a mutagenicity endpoint, although the percentage of prediction accuracy (probability score) was low (52%).

### 2.5. MD Simulation Analysis

**Figure 4:**
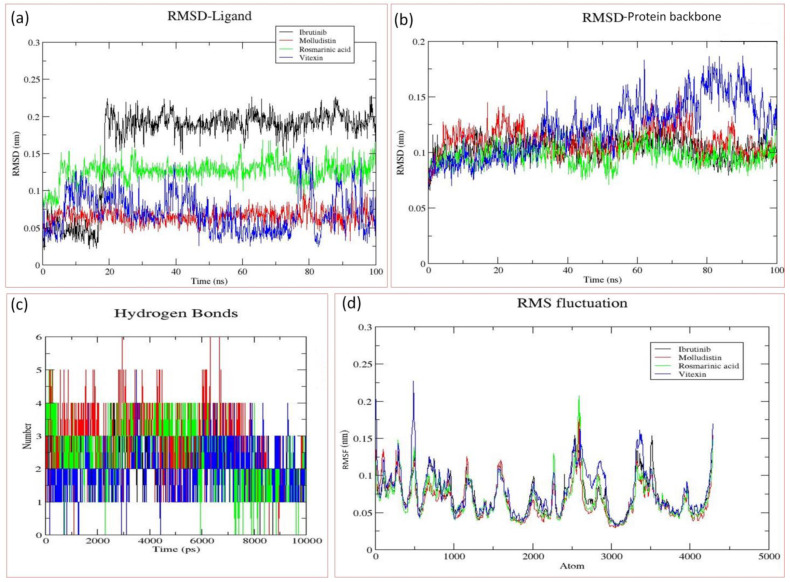
Molecular dynamics (MD) analysis of the docking complexes of the best three phytochemicals (Molludistin, rosmarinic acid, and Vitexin) and the control drug (Ibrutinib). (**a**) RMSD of different ligands in the binding pocket of Bruton’s tyrosine kinase (BTK), (**b**) RMSD of the protein (BTK) backbone, (**c**) Hydrogen bond plots of the different ligands with BTK, and (**d**) RMSF of the atoms of BTK backbone when bound with different ligands. The ligands, their corresponding protein backbones, and their hydrogen bond pattern are uniformly color coded in the figure (Ibrutinib, in black; Molludistin, in red; rosmarinic acid, in green; and Vitexin, in blue).

After screening of the 46 phytochemicals of *O. sanctum* through in silico methods, including molecular docking, ADME analysis, and in silico toxicity assay, three compounds (Molludistin, rosmarinic acid, and Vitexin) showed promising results and were selected for further analysis through molecular dynamics (MD) simulations. In MD simulations, an estimate of the stability and dynamic nature of the protein–ligand complex is estimated by measuring the RMSD of ligand and the protein backbone in the complex. Here, we report the RMSD of BTK backbone and the ligands (Molludistin, rosmarinic acid, and Vitexin) in their respective docking complexes. Interestingly, on MD analysis of the three selected compounds, Molludistin revealed a highest degree of stability, followed by Rosmarinic acid, with least structural deviations (less than 1 Å) in their corresponding docking complexes with BTK (Figure 4a). However, the compound, Vitexin, was found to be relatively less stable in its docking complex, showing higher deviations (slightly greater than 1 Å) (Figure 4a). It is worth mentioning here that the RMSD fluctuations of all the three compounds were within the acceptable limit of 2.0 Å, suggesting that the docking complexes of all the three phytochemicals (Molludistin, rosmarinic acid, and Vitexin) are stable. The protein backbone of BTK was also observed to be stable while bound to Molludistin and rosmarinic acid in their corresponding docking complexes, as indicated by the low RMSD of less than 1 Å (Figure 4b). However, relatively higher RMSD (slightly greater than 1 Å) was observed in the BTK protein backbone while in complex with Vitexin (Figure 4b). The hydrogen bonds of these ligands with the active site residues of BTK during MD analysis are represented in Figure 4c. Furthermore, the local conformational changes in the side chains of the target protein on binding a particular ligand can be determined by analyzing the RMSF of the target protein. In the current study, the RMSF of BTK on binding with the selected ligands (Molludistin, rosmarinic acid, and Vitexin) was also analyzed and reported (Figure 4d). The results indicate that the RMSF of BTK did not fluctuate too much and remained within the acceptable limits, thereby demonstrating that the overall conformation of target protein was conserved during/after ligand binding in all complexes. 

Based on our observations, we report here that among all the 46 phytochemicals of *O. sanctum*, the three compounds (Molludistin, Rosmarinic acid, and Vitexin) are the best binders and possible inhibitors of BTK. However, further studies are warranted to confirm these findings.

## 3. Discussion

Bruron’s tyrosine kinase (BTK) is a member of the Tec family tyrosine kinase and is expressed in both normal as well as in malignant B-cells [28]. The inhibition of BTK has become the goal of the treatment of B-cell malignancies, including chronic lymphocytic leukemia (CLL). Some BTKIs (Ibrutinib, Acalabrutinib, and Zanubrutinib) are currently available in the market and are being used in CLL therapy. BTKIs, covalently, and irreversibly, bind to cysteine 481 of BTK, thereby inhibiting the enzyme [29]. However, due to the variable treatment outcomes, drug resistance, and side effects of the currently available drugs for CLL, new drugs with improved efficacy, reduced drug resistance, and no or relatively low side effects are required for more efficient treatment of the disease. Keeping in mind the limitations of the available BTKIs, we focused on the possibility of using herbal products as alternate BTKIs for the CLL treatment. In this preliminary study, we selected the phytochemicals of *O. sanctum*, a well-used herb in traditional medicine since times immemorial. The phytochemicals of *O. sanctum* were screened for their binding and inhibitory potential towards BTK through in silico procedures. In the initial screening by molecular docking procedure, six compounds (Vicenin-2, Luteolin-7-O-glucuronide, Molludistin, Galuteolin, Vitexin, and rosmarinic acid), among the 46 selected phytocompounds, showed a significant binding affinity with BTK, as revealed by their higher docking scores/lower free energy (ΔG) values (−9.2 kcal/mol to −10 kcal/mol). The docking scores of these six top-ranked phytochemicals (Vicenin-2, Luteolin-7-O-glucuronide, Molludistin, Galuteolin, Vitexin, and rosmarinic acid) were comparable to those of the standard BTKIs/control drugs, Acalabrutinib (−10.3 kcal/mol) and Ibrutinib (−11.3 kcal/mol) (see Table 1). The docking results were confirmed by redocking Ibrutinib (extracted from PDB protein structure, 5P9J) with the empty BTK protein. The grid dimensions for docking and redocking were selected, such that the active site of BTK is occupied in the grid box [30]. On visualizing the different poses of phytochemicals in their docking complexes with BTK, all the six top-ranked phytochemicals were observed to bind in the active site of BTK, as observed for control dugs (Figure 1). The interaction studies showed that these compounds interacted with the various active site residues through different types of interactions, including hydrogen bonding, hydrophobic interactions, and others (see Figure 3). As far as hydrogen bonding is concerned, Galuteolin, Luteolin-7-O-glucuronide, Molludistin, Rosmarinic acid, Vicenin-2, and Vitexin interacted with active site residues of BTK through 7, 7, 5, 5, 6, and 2 hydrogen bonds, respectively (Figure 3). The details of the different bond types, their bond lengths, and the interacting residues in the active site of BTK are shown in Figure 2 for control drugs/standard BTKIs, and in Figure 3 for the six top-ranked phytochemicals of *O. sanctum*.

The inhibitory potential of an inhibitor towards an enzyme or a protein receptor does not assure its suitability as an efficient drug [31]. The ADME analysis of the inhibitory compounds has been considered critical in drug discovery, as it helps in making the correct decision regarding the selection of inhibitors for evaluation in a living system [31,32,33]. Therefore, in the present study, the ADME analysis of the six top-ranked phytochemicals was performed for further screening, and selection of the best compounds possessing drug-like characteristics (Table 2; Supplementary Table S1). Moreover, during ADME analysis, the Lipinski rule of 5 is broadly considered while selecting the drug-like characteristics of a compound [34,35,36]. Based on Lipinski rule of 5 and other ADME analysis filters, three phytocompounds, viz., Molludistin, rosmarinic acid, and Vitexin possessed promising drug-like characteristics (Table 2; Supplementary Table S1). On performing the in-silico toxicity assay of the three selected compounds (Molludistin, Rosmarinic acid, and Vitexin), it was observed that all the three compounds are less toxic and relatively safe, with their toxicity level/class being either equal to or less than that of the standard BTKI, ibrutinib (Table 3; Supplementary Table S2). Furthermore, on MD analysis of the three selected compounds, Molludistin revealed a highest degree of stability, followed by Rosmarinic acid, and Vitexin, with least structural deviations in their corresponding docking complexes with BTK. The stability of Molludistin, Rosmarinic acid, and Vitexin in the binding pocket of the BTK was found to be more than that of the standard BTKI (Ibrutinib) (Figure 4a). Moreover, the hydrogen bond plot indicated a higher frequency of bond formation by the compounds Molludistin and Rosmarinic acid compared to ibrutinib, which may be attributed to its higher stability and potency in the binding pockets of BTK. Our observations indicate that the three compounds (Molludistin, Rosmarinic acid, and Vitexin) possessing drug likeliness characteristics, stably bind and inhibit BTK and can be therefore considered as possible promising inhibitors of BTK. However, further evaluation of these compounds through experimental analysis is warranted to confirm the in-silico observations reported in this study.

## 4. Materials and Methods

### 4.1. Retrieval of Protein and Ligand Structures

The crystal structure of the protein BTK in complex with the inhibitor ibrutinib (PDB ID: 5P9J) was retrieved from the RCSB protein data bank (http://www.rscb.org (accessed on 25 February 2023)), whereas the three-dimensional conformers of the selected phytochemicals and the control inhibitors (ibrutinib and acalabrutinib) were downloaded from PubChem (https://pubchem.ncbi.nlm.nih.gov/ (accessed on 24 February 2023)). The protein and ligands (phytochemicals and the control inhibitors) were downloaded and saved in PDB format and SDF format, respectively [37].

### 4.2. Preparation of Protein and Ligands for Docking

The PDB structure of BTK and ligands were prepared for molecular docking using UCSF Chimera 1.12 software, as reported earlier [37]. Briefly, the protein preparation mainly involved the removal of water molecules and the hetero-atoms and addition of polar hydrogens and appropriate charges. The ligand preparation was carried out by setting the torsion roots, the addition of gasteiger charges, and the assimilation of non-polar hydrogens [37].

### 4.3. Molecular Docking Procedure and Interaction Studies

The dimensions of the grid box surrounding the active site residues of BTK were searched from the literature and used in our docking procedure [30]. The molecular docking was performed using Autodock Vina embedded in UCSF Chimera 1.12 software [38]. The grid dimensions of BTK (in Å) for active site-specific docking were fixed at x: 24, y: 28, z: 32 [30]. Ibrutinib and acalabrutinib were used as a positive control in the docking procedure. The ligand (ibrutinib) in the original PDB protein structure 5P9J was extracted from the structure and redocked with the empty protein to validate the docking procedure. The docking complexes were made using PyMol molecular graphics system and the interaction of the ligands with active site residues of 5P9J were analysed using BioVia Discovery Studio, as reported earlier [37,39,40].

### 4.4. ADME Analysis of Selected Phytochemicals

The analysis of the absorption, distribution, metabolism, and excretion (ADME) of the compounds has been reported very critical in drug discovery [31,32,33]. Therefore, the ADME study was conducted to analyse the drug-likeness, pharmacokinetics, and physicochemical properties of the selected phytochemicals using the web-based Swiss ADME tool (http://www.swissadme.ch (accessed on 2 March 2023)) [41].

### 4.5. In Silico Toxicity Prediction

The ProTox-II (http://tox.charite.de/protox_II/ (accessed on 20 March 2023)), a free web server, was used for predicting the toxicity of selected phytochemicals and the control drug (ibrutinib). This tool represents a novel method in toxicity prediction [42]. The toxic doses for oral toxicity are expressed as LD50 values in milligrams per Kg body weight (mg/Kgbw), depending on which the compounds can be classified into different toxicity classes different toxicity classes, which are defined according to the Globally Harmonized System (GHS) of classification for labelling of chemicals [43].

### 4.6. Molecular Dynamics Simulations

The molecular dynamics (MD) simulations of the protein–ligand complexes of the selected top-ranked phytochemicals/ligands were performed using GROMACS software version 2020 [44]. The CHARMM36 updated 2020 force field [45] was used for MD simulations. Complexes were solvated in cubic water boxes containing transferable intermolecular potential with three points (TIP3P) water molecules. The system was neutralized by adding ions using “genion” module of gmx. The CGenFF server [46] was used to construct the ligand topology. The system began by doing 10,000 steps of the sharpest fall while minimizing energy use. After that, the minimized system was used to perform simulations with a NVT and NPT ensemble using the minimized system. After the two equilibration stages were completed, a MD simulation at 100 ns was performed. Temperature was not included in this simulation. All the generated trajectories were examined for conformational deviations once the MD simulation was completed. The stability and flexibility of the protein and ligand were determined using the root mean square deviation (RMSD), RMSF, and hydrogen bonding.

## 5. Conclusions

In this study, the phytochemicals of *O. sanctum* were screened for their potential to bind and inhibit Bruton’s tyrosine kinase (BTK). This preliminary study was performed using various in silico screening procedures, including the molecular docking, ADME analysis, toxicity assay, and the MD simulations. The molecular docking observations revealed that out of the 46 phytochemicals of *O. sanctum*, six compounds (Vicenin-2, Luteolin-7-O-glucuronide, Molludistin, Galuteolin, Vitexin, and Rosmarinic acid) possessed significantly higher docking scores, which were comparable to those of the control inhibitors, acalabrutinib and ibrutinib. However, ADME analysis of these six compounds revealed that only three compounds (Molludistin, Rosmarinic acid, and Vitexin) possessed drug likeliness characteristics, and they are relatively less toxic and safe as revealed by the in silico toxicity prediction assay. Moreover, the three compounds (Molludistin, Rosmarinic acid, and Vitexin), were found to remain stable during the MD analysis. Therefore, in conclusion, the findings of our study suggest that Molludistin, Rosmarinic acid, and Vitexin, which possess the good characteristics for inhibiting BTK, could be the potential molecules to be considered in the ongoing drug discovery strategies for the treatment of CLL. However, our in-silico observations need further confirmation through in vitro and in vivo studies.

## Figures and Tables

**Table 1:** Compound ID and molecular weight of the phytochemicals of *Ocimum sanctum*, and their corresponding docking scores after molecular docking with Bruton’s tyrosine kinase (BTK).

S. No	Name of Phytochemical/Ligand	Compound ID (CID)	Molecular Weight (g/mol)	Docking Score (kcal/mol)
1	3-carene	CID_26049	136.23	−6.7
2	4-hydroxybenzaldehyde	CID_126	122.12	−5.6
3	4-hydroxybenzoic acid	CID_135	138.12	−5.9
4	Aesculin	CID_5281417	340.28	−7.9
5	Alpha-Cadinol	CID_10398656	222.37	−7.3
6	Ascorbic acid	CID_54670067	176.12	−5.6
7	Bergamotene	CID_521569	204.35	−7.2
8	Cadinene	CID_441005	204.35	−7.2
9	Carvacrol	CID_10364	150.22	−6.2
10	Caryophyllene	CID_5281515	204.35	−6.7
11	Chlorogenic acid	CID_1794427	354.31	−8.4
12	Cirsilineol	CID_162464	344.3	−8.7
13	Cirsimaritin	CID_188323	314.29	−8.7
14	Citral	CID_638011	152.23	−6.0
15	Estragole	CID_8815	148.20	−6.1
16	Eucalyptol	CID_2758	154.25	−4.8
17	Eugenol	CID_3314	164.20	−5.9
18	Gallic acid	CID_370	170.12	−6.1
19	Gallic acid ethyl ester	CID_13250	198.17	−6.3
20	Gallic acid methyl ester	CID_7428	184.15	−6.2
21	Galuteolin	CID_5280637	448.4	−9.6
22	Isorientin	CID_44257986	730.6	−8.5
23	Isothymonin	CID_11726019	360.3	−8.4
24	Isothymusin	CID_630253	330.29	−8.6
25	Isovitexin	CID_162350	432.4	−8.9
26	Linoleic acid	CID_5280450	280.4	−5.7
27	Linolenic acid	CID_5280934	278.4	−6.7
28	Luteolin	CID_5280445	286.24	−8.9
29	Luteolin−7-O-glucuronide	CID_13607752	462.4	−9.8
30	Methyl cinnamate	CID_637520	162.18	−6.5
31	Methyl eugenol	CID_7127	178.23	−5.9
32	Methylchavicol	CID_8815	148.20	−5.7
33	Molludistin	CID_44258315	416.4	−9.7
34	Ocimene	CID_5281553	136.23	−5.7
35	Oleic acid	CID_445639	282.5	−6.0
36	Rosmarinic acid	CID_5281792	360.3	−9.2
37	Sitosterol	CID_222284	414.7	−8.5
38	Stearic acid	CID_5281	284.5	−5.9
39	Terpinene-4-ol	CID_11230	154.25	−5.5
40	Ursolic acid	CID_64945	456.7	−8.9
41	Vanillic acid	CID_8468	168.15	−6.0
42	Vicenin	CID_3084407	594.5	−8.9
43	Vicenin-2	CID_442664	594.5	−10.0
44	Vitexin	CID_5280441	432.4	−9.3
45	Alpha-pinene	CID_6654	136.23	−7.2
46	Beta- pinene	CID_14896	136.23	−6.7
Ibrutinib (Control drug)		CID_24821094	440.5	−11.3
Acalabrutinib (Control drug)		CID_71226662	465.5	−10.3

**Table 2:** Physicochemical properties, drug-likeliness, and water solubility of the top-ranked phytochemicals of *O. sanctum*.

Tested Phytochemicals	Physicochemical Properties							Drug-Likeliness		Water Solubility	
	MW	HA	RB	HBA	HBD	MR	TPSA	L-V	BS	Log S (ESOL)	Log S (Ali)
Galuteolin	448.38	32	4	11	7	108.13	190.28 Å^2^	2	0.17	Soluble	Moderately soluble
Luteolin-7-O-glucuronide	462.36	33	4	12	7	108.74	207.35 Å^2^	2	0.11	Soluble	Moderately soluble
Molludistin	416.38	30	3	9	5	105.11	149.82 Å^2^	0	0.55	Soluble	Soluble
Rosmarinic acid	360.31	26	7	8	5	91.4	144.52 Å^2^	0	0.56	Soluble	Moderately soluble
Vicenin-2	594.52	42	5	15	11	139.23	271.2 Å^2^	3	0.17	Soluble	Soluble
Vitexin	432.38	31	3	10	7	106.61	181.05 Å^2^	1	0.55	Soluble	Soluble

MW (molecular weight), HA (number of heavy atoms), RB (number of rotatable bonds), HBA (number of hydrogen bond acceptors), HBD (number of hydrogen bond donors), MR (molar refractivity), TPSA (topological polar surface area), L-V (Lipinski’s rule violation), and BS (bioavailability score).

**Table 3:** Oral toxicity prediction of selected phytochemicals and the control drug using ProTox-II web server.

Tested Phytochemicals	Results of Oral Toxicity Prediction			
	Predicted Toxicity Class	Predicted LD50 (mg/Kgbw)	Average Similarity (%)	Prediction Accuracy (%)
Molludistin	Class IV	832	60.24	68.07
Rosmarinic acid	Class V	5000	63.44	68.07
Vitexin	Class IV	832	58.71	67.38
Ibrutinib (control drug)	Class IV	1000	48.69	54.26

Class I: fatal if swallowed (LD50 ≤ 5 mg/Kgbw); Class II: fatal if swallowed (5 mg/Kgbw < LD50 ≤ 50 mg/Kgbw); Class III: toxic if swallowed (50 mg/Kgbw < LD50 ≤ 300 mg/Kgbw); Class IV: harmful if swallowed (300 mg/Kgbw < LD50 ≤ 2000 mg/Kgbw); Class V: may be harmful if swallowed (2000 mg/Kgbw < LD50 ≤ 5000 mg/Kgbw).

## Data Availability

The data presented in this study are available in the manuscript itself and in the associated supplementary information.

## Supplementary Materials

The following supporting information can be downloaded at: https://www.mdpi.com/article/10.3390/molecules28083287/s1, Table S1. SwissADME analysis report of the six top-ranked phytochemicals of *O. sanctum*. Table S2. Hepatotoxicity and toxicological endpoint predictions estimated using the ProTox-II web server. Figure S1. Three-dimensional (3D) illustration of the binding poses of the top-ranked phytochemicals of *O. sanctum* and the control drug (ibrutinib) to the active site of Bruton’s Tyrosine Kinase (PDB ID: 5P9J). The ligands (phytochemicals and ibrutinib) are represented in spheres. (a) Ibrutinib (Limegreen), (b) Galuteolin (Yellow), (c) Luteolin-7-O-glucuronide (Blue), (d) Molludistin (Magenta), (e) Rosmarinic acid (Red), (f) Vicenin-2 (Cyan), (g) Vitexin (Orange).
