# Supplementary material for: Inhibitory Potential of the Ocimum sanctum Phytochemicals on Bruton’s Tyrosine Kinase, a Well-Known Drug Target for Treatment of Chronic Lymphocytic Leukemia: An In Silico Investigation
Source: Molecules. 2023 Apr 7;28(8):3287. doi: 10.3390/molecules28083287 (PMC10144307; doi:10.3390/molecules28083287)
Supplement: Supplementary file 1 [file molecules-28-03287-s001.zip › molecules-2298265-supplementary.pdf]

Title: Inhibitory potential of the *Ocimum sanctum* phytochemicals on Bruton's Tyrosine Kinase, a well-known drug target for treatment of Chronic Lymphocytic Leukemia: An *in-silico* investigation

Shabir Ahmad Mir [1, 2 *], Yahya Madkhali [1], Ahmad Firoz [3], Ayoub Al Othaim [1], Wael Alturaiki [1], Sami G. Almalki [1], Abdulrahman Algarni [4], Suliman A Alsagaby [1]

[1] Department of Medical Laboratory Sciences, College of Applied Medical Science, Majmaah University, Al Majmaah-11952, Saudi Arabia.

[2] Health and Basic Sciences Research Center, Majmaah University, Al Majmaah 11952, Saudi Arabia

[3] Department of Biological Sciences, Faculty of Science, King Abdulaziz University, Jeddah, Saudi Arabia.

[4] Department of Medical Laboratory Technology, Faculty of Applied Medical Sciences, Northern Border University, Arar 91431, Saudi Arabia.

*** Address for correspondence**

Dr Shabir Ahmad Mir

Assistant Professor

Department of Medical Laboratory Sciences,

College of Applied Medical Science,

Majmaah University, Saudi Arabia.

Phone: +966536300645

Email: s.mir@mu.edu.sa

Supplementary Tables:

Supplementary Table S1. SwissADME analysis report of the six top-ranked phytochemicals of O. sanctum.

ADME Characteristics		Phytochemicals					
		Galuteolin	Luteolin-7-O-glucuronide	Molludistin	Rosmarinic acid	Vicenin-2	Vitexin
Physicochemical properties	Molecular weight (g/mol)	448.38	462.36	416.38	360.31	594.52	432.38
	No. of heavy atoms	32	33	30	26	42	31
	No. of arom. heavy atoms	16	16	16	12	16	16
	No. of rotatable bonds	4	4	3	7	5	3
	No. of H-bond acceptors	11	12	9	8	15	10
	No. of H-bond donors	7	7	5	5	11	7
	Molar Refractivity	108.13	108.74	105.11	91.4	139.23	106.61
	TPSA	190.28 Å²	207.35 Å²	149.82 Å²	144.52 Å²	271.2 Å²	181.05 Å²
Lipophilicity	Log Po/w (iLOGP)	1.76	1.56	2.2	1.48	1.27	1.63
	Log Po/w (XLOGP3)	1.46	0.97	0.6	2.36	-2.26	0.21
	Log Po/w (WLOGP)	-0.24	-0.15	0.71	1.65	-3.04	-0.23
Water Solubility	Log S (ESOL)	-3.65	-3.41	-3	-3.44	-2.05	-2.84
	Class	Soluble	Soluble	Soluble	Soluble	Soluble	Soluble
	Log S (Ali)	-5.06	-4.91	-3.32	-5.04	-2.9	-3.57
	Class	Moderately soluble	Moderately soluble	Soluble	Moderately soluble	Soluble	Soluble
Pharmacokinetics	GI absorption	Low	Low	Low	Low	Low	Low
	BBB permeant	No	No	No	No	No	No
	P-gp substrate	Yes	Yes	No	No	No	No
	CYP1A2 inhibitor	No	No	No	No	No	No
	CYP2C19 inhibitor	No	No	No	No	No	No
	CYP2C9 inhibitor	No	No	No	No	No	No
	CYP2D6 inhibitor	No	No	No	No	No	No
	CYP3A4 inhibitor	No	No	No	No	No	No
	Log Kp (skin permeation)	-8.00 cm/s	-8.43 cm/s	-8.41 cm/s	-6.82 cm/s	-11.53 cm/s	-8.79 cm/s
Drug-likeness	Lipinski	No; 2 violations: NorO>10, NHorOH>5	No; 2 violations: NorO>10, NHorOH>5	Yes; 0 violation	Yes; 0 violation	No; 3 violations: MW>500, NorO>10, NHorOH>5	Yes; 1 violation: NHorOH>5
	Ghose	Yes	Yes	Yes	Yes	No; 4 violations: MW>480, WLOGP <-0.4, MR>130, #atoms>70	Yes
	Bioavailability Score	0.17	0.11	0.55	0.56	0.17	0.55
Medicinal Chemistry	PAINS	1 alert: catechol_A	1 alert: catechol_A	0 alert	1 alert: catechol_A	0 alert	0 alert
	Brenk	1 alert: catechol	1 alert: catechol	0 alert	2 alerts: catechol, michael_acceptor_1	0 alert	0 alert
	Synthetic accessibility	5.17	5.11	4.91	3.38	6.40	5.12

Supplementary Table S2. Hepatotoxicity and toxicological endpoint predictions estimated using the ProTox-II web server.

Phytochemicals	**Organ Toxicity (% Probability)**				
	**Organ Toxicity (% Probability)**	**Toxicity Endpoint (% Probability)**			
	**Hepatotoxicity**	**Carcinogenicity**	**Immunotoxicity**	**Mutagenicity**	**Cytotoxicity**
Molludistin	Inactive (81)	Inactive (78)	Active (82)	Inactive (67)	Inactive (71)
Rosmarinic acid	Inactive (62)	Inactive (66)	Active (93)	Inactive (85)	Inactive (90)
Vitexin	Inactive (81)	Inactive (72)	Inactive (82)	Active (52)	Inactive (87)
Ibrutinib (Control drug)	Inactive (69)	Active (58)	Inactive (66)	Inactive (54)	Inactive (66)

Supplementary Figures:

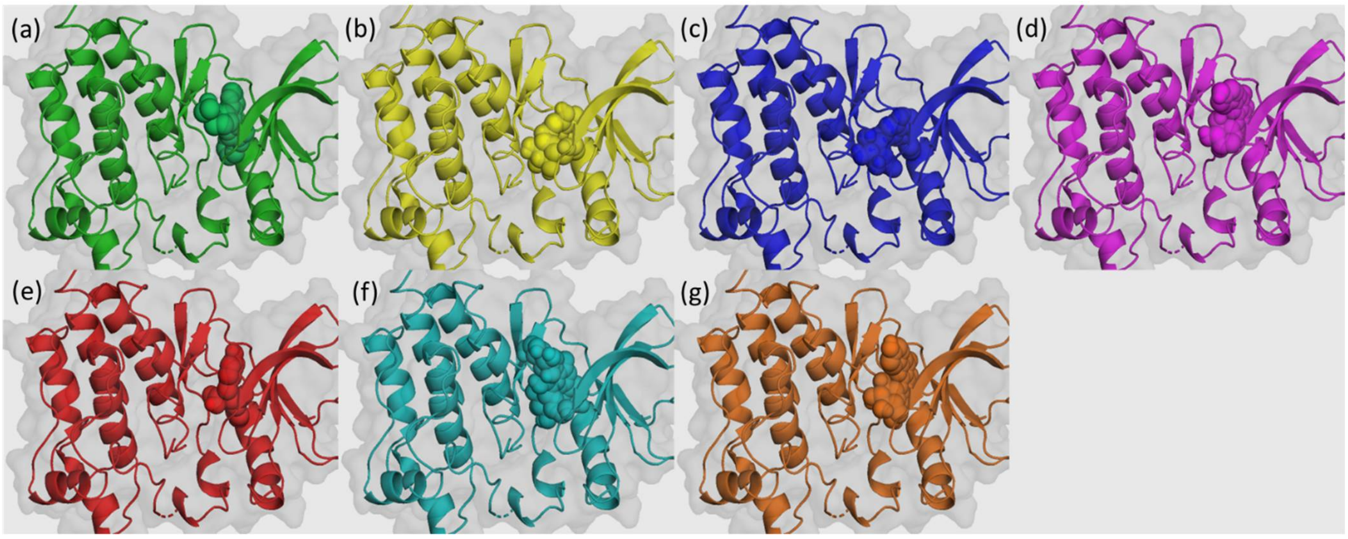

Supplementary Figure S1. Three-dimensional (3D) illustration of the binding poses of the top-ranked phytochemicals of ***O. sanctum*** and the control drug (ibrutinib) to the active site of Bruton's Tyrosine Kinase (PDB ID: 5P9J). The ligands (phytochemicals and ibrutinib) are represented in spheres. (a) Ibrutinib (Limegreen), (b) Galuteolin (Yellow), (c) Luteolin-7-O-glucuronide (Blue), (d) Molludistin (Magenta), (e) Rosmarinic acid (Red), (f) Vicenin-2 (Cyan), (g) Vitexin (Orange).
